# PICC-PORT: Valid indication to placement in patient with results of extensive skin burns of the neck and chest in oncology. The first case in the scientific literature

**DOI:** 10.1016/j.ijscr.2020.02.028

**Published:** 2020-02-19

**Authors:** D. Merlicco, M. Lombardi, M.C. Fino

**Affiliations:** a Vascular Access Center - General Surgery Unit, University Polyclinic Foggia-Lucera (Fg), Italy; b Oncology Operative Unit, San Severo Hospital (Fg), Italy; c Clinical Oncology, University Polyclinic Foggia - Lucera (Fg), Italy

**Keywords:** PICC-PORT, Brachial port, Totally implantable central venous access, Chemotherapy

## Abstract

**Introduction:**

The management of patients with advanced gastric cancer requires a stable venous access required at different stages of disease (treatment phase, palliative phase). Totally implantable central venous access in the arm, named PICC-PORT, is used in a patient with results of extensive skin burns of the neck, chest and right arm and surgical outcomes of multiple skin grafts of chest. The described clinical case is the first event in the scientific literature.

**Presentation of case:**

We report a case of a patient with results of extensive skin burns of the neck, chest and right arm and surgical outcomes of multiple skin grafts of chest that must perform chemotherapy with a port. In this patient, due to the difficulty of implanting a door in the cervico-thoracic district, we opt for the totally subcutaneous implantation of the vascular device (PICC-PORT 5 Fr) in the left arm.

**Discussion:**

In this patient for the difficulty of implanting a port in the cervico-thoracic district, we opt for the totally subcutaneous implantation of the vascular device (PICC-PORT) in the arm. The PICC-PORT is positioned with the same technique as the PICC (ultrasound-guided vein puncture, with modified Seldinger technique and tip location with ECG technique); presents all the functional and aesthetic advantages of a totally subcutaneous device.The case described is the first case in the scientific literature.

**Conclusion:**

In Europe in recent years for the availability of the vascular device of small dimensions and materials increasingly compatible, the "PICC-PORT" is positioned in the veins of the arm with ultrasound-guidance without complications such as pneumothorax, arterial puncture, hematoma of the neck, Pinch-off syndrome, such as the clinical case presented with extensive scars on the chest and neck. Thoracic devices (chest port, tunnel venous catheter) are not indicated in thickened and inelastic skins, due to the high risk of dehiscence of the surgical wound.

## Highlights

• We report a case of cancer patient with extensive skin burns to the neck-chest-arm.
• PICC-PORT is the only Device ensure safe infusions of chemotherapy.
• PICC-PORT are placed in the veins of the arm with ultrasound-guidance.
• PICC-PORT 5Fr was implanted in the brachial vein for the neck-chest skin grafts.
• PICC-PORT is free of complications such as pneumothorax, Pinch-off syndrome.

## 1 Introduction

The management of patients with advanced gastric cancer requires a stable venous access required at different stages of disease (diagnostic phase, treatment phase, palliative phase) [1]. Totally implantable central venous access (Port) is often used for ensure safe infusions of chemotherapy or pain management and supportive care in cancer patients. Local and systemic complications may occur both during and after placement of port despite the well-established techniques for its placement and care.

Incidence and nature of complications of central venous catheter have been well established for long-term chemotherapy. However, very sparse data exists on the incidence of complications of molecularly targeted therapies administered through a totally implantable central venous access [2].

Retrospectively analyzing patients undergoing conventional chemotherapy, we report a case of a patient with results of extensive skin burns of the neck, chest and right arm and surgical outcomes of multiple skin grafts of chest to which a “PICC-PORT” was implanted in the left brachial vein [[3], [4], [5], [6]].

In this patient for the difficulty of implanting a port in the cervical-thoracic district, we recommend the totally subcutaneous implantation of the vascular device with peripheral access to the left arm, the PICC-PORT; other indications in our experience are head and neck cancer, patients with Radiodermatitis by radiation therapy for head and neck cancer, patients with ostomies (tracheobronchial/esophagus-ostomy), patients in molecular targeted therapy (EGFR-i), patients with severe kyphosis, patients with breast implants bilaterally, is added to the patient with extensive burns to the neck and chest or further skin grafts.

In Europe in recent years, for the availability of the vascular device of small dimensions and materials increasingly compatible, are implanting "PICC-PORT"; they are placed in the veins of the arm with ultrasound-guidance and the system appears to be free of early complications such as pneumothorax, arterial puncture, hematoma of the neck, pinch off syndrome [7].

This work has been reported in line with the SCARE criteria [8].

## 2 Presentation of case

The patient is a 45-year-old man, in the November 2015 found during clinical-instrumental tests an advanced gastric cancer and subjected to Gastrectomy Subtotal with Billroth II reconstruction; in December 2015 comes to our observation in the DH of Oncology to undergo adjuvant chemotherapy. The oncologist, in anticipation of a combination chemotherapy with ECF (Epirubicin + Cisplatin + 5-FU), requires a stable central venous access for advanced disease.

**Image 1:**
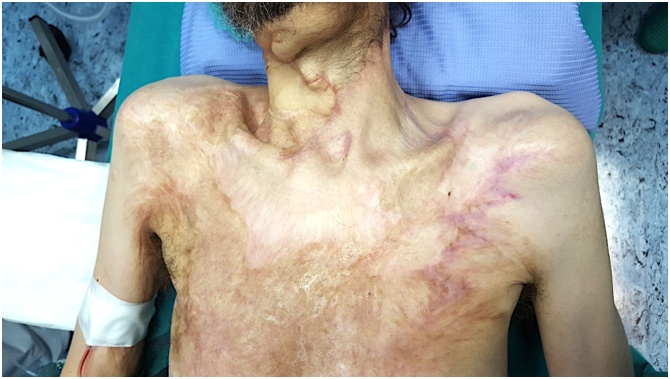
Results of extensive skin burns of the neck, chest and right arm and surgical outcomes of multiple skin grafts of chest.

Comes to our Vascular Access Ambulatory, the patient's medical examination showed the scars from a previous large burn of the neck, chest and right arm and surgical outcomes of multiple skin grafts chest (Image 1).

The difficulty of implanting a port in the cervical-thoracic district, we opt for the totally subcutaneous implantation of the vascular device in the left arm.

**Image 2:**
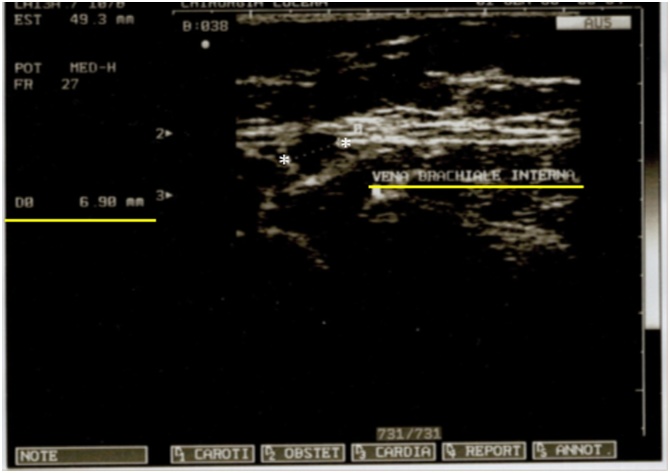
Ultrasound of arm veins; diameter of the brachial vein of the left arm.

**Image 3:**
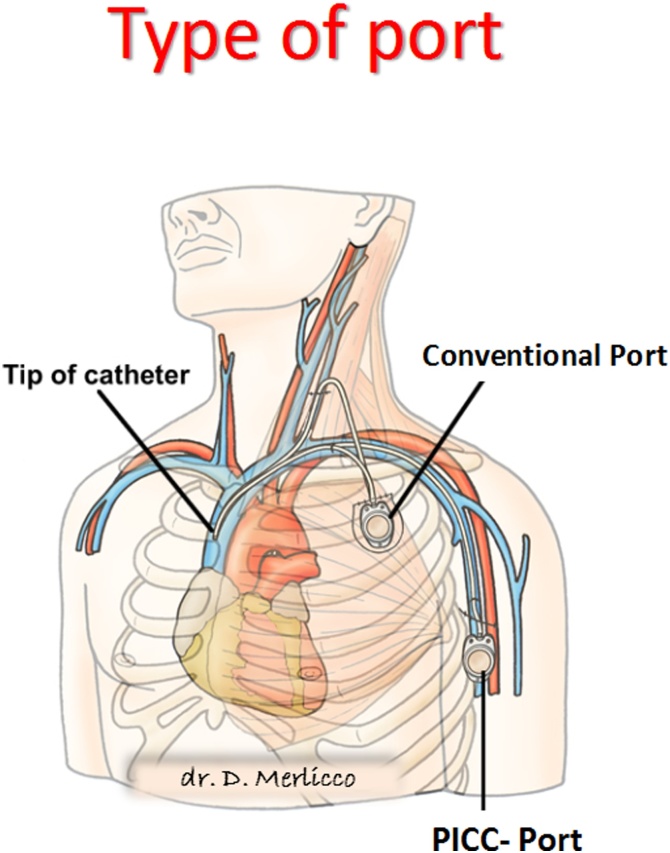
Type of port: Conventional Port and Picc-Port.

We performed an US of the left arm to assess the diameters of the brachial veins and basilica; it detects an internal brachial vein of good caliber 6,9 mm and you decide to implant a “PICC-PORT” (Images 2 and 3).

**Image 4:**
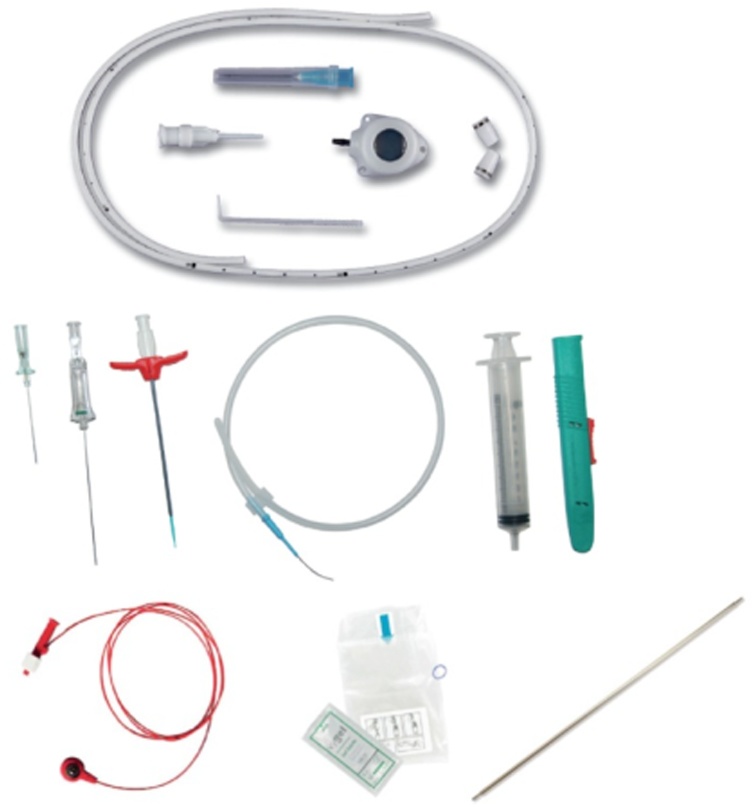
Device 5 Fr used for placement of Picc-Port.

The device used for the plant was a Brachial Micro-Port 5 Fr, PUR, non-valved catheter, with hybrid reservoir (Titanium e Polyoxymethylene), in internal brachial vein left with ultrasound/ECG guidance (Image 4).

## 3 Discussion

**Image 5:**
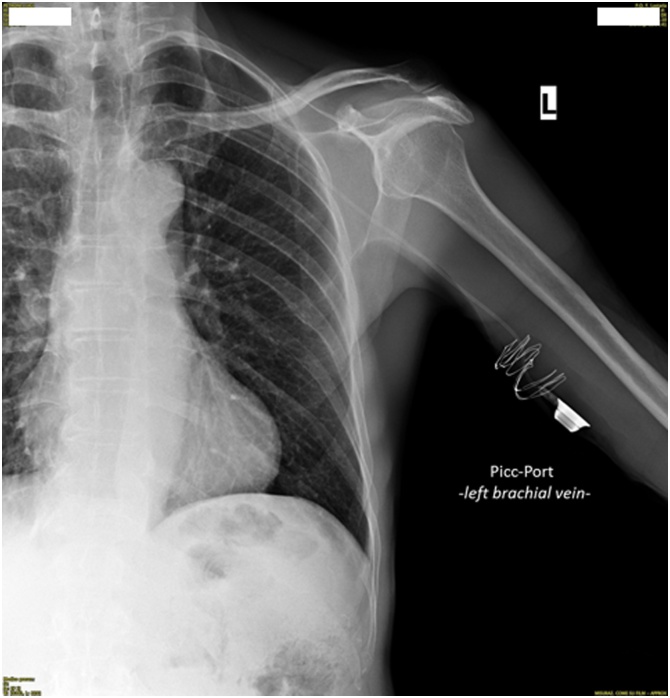
Post-procedural chest radiography.

The implantation of PICC-PORT was performed in patient with results of extensive skin burns of the chest; the catheter was placed in arm without skin burns; the vein of choice for US-guided puncture was the internal brachial. There were no early postoperative complications (hematoma, bleeding, displacements, rotations, detachment of the CVC from the reservoir) (Image 5).

**Image 6:**
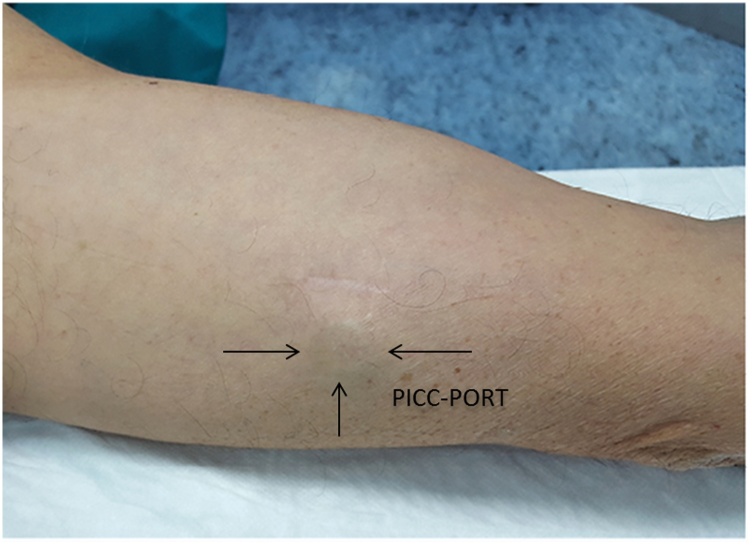
Picc-Port to the left arm, 365 days after implant.

The outcomes at 395 days (corresponds to the patient's death) after implantation is optimal, it was not complicated by thrombotic or infectious phenomena and the patient is satisfied in terms of aesthetic and social reintegration (Image 6).

## 4 Conclusion

The PICC-PORT is a Vascular Device, used in recent years, for the administration of Poly-Chemotherapies or Parenteral Nutrition (NP) or Chronic Pain Therapies; it is a device made up of a totally implantable brachial chamber and a central venous catheter in PUR (polyurethane) or SIL (silicone).

The PICC-PORT is positioned with the same technique as the PICC (ultrasound-guided vein puncture, with modified Seldinger technique and tip location with ECG technique); presents all the functional and aesthetic advantages of a totally subcutaneous device.

In the illustrated clinical case, the patient has extensive scars on the chest and neck, therefore the thoracic devices (chest Port, Tunneled venous catheter) cannot be implanted for thickened, inelastic skin, with a high risk of dehiscence of the surgical wound.

The case described is the first case in the scientific literature.

Implantation of the PICC-PORT is indicated in patient with head and neck cancer, patients with Radiodermatitis by radiation therapy for head and neck cancer, patients with ostomies (tracheobronchial/esophagus-ostomy), patients in molecular targeted therapy (EGFR-i), patients with severe kyphosis, patients with breast implants bilaterally, but with this case-report we can say even in patient with extensive burns to the neck, chest or further skin grafts.

The PICC-PORT is positioned in the veins of the arm with ultrasound-guidance and the system appears to be free of early complications such as pneumothorax, arterial puncture, hematoma of the neck, Pinch-off syndrome.

## Sources of funding

None.

## Ethical approval

The ethical approval has been exempted by my institution.

## Consent

Written informed consent was obtained from the patient for publication of this case report and accompanying images. A copy of the written consent is available for review by the Editor-in-Chief of this journal on request.

## Author contributions

1. Dr. Domenico Merlicco, Chief of the Vascular Access Center - Division of Surgery, University Polyclinic Foggia - Lucera (Fg), Italy: 50% contributed to the manuscript.
2. Dr. Massimo Lombardi, Oncology Operative Unit, San Severo Hospital (Fg), Italy: 25% contributed to the manuscript.
3. Dr. Maria Carmela Fino, Clinical Oncology Nurse, University Polyclinic Foggia - Lucera (Fg), Italy: 25% contributed to the manuscript.

## Registration of research studies

All the authors have made substantial contributions to all the following aspects: (1) study design and planning, data acquisition, data analysis and interpretation, (2) article writing or critical review of important intellectual contents (3) approval final version to be presented. All authors have read and approved the manuscript for publication.

## Guarantor

Domenico Merlicco MD, Chief of the Vascular Access Center, University Polyclinic Foggia - Lucera (Fg), Italy.

## Provenance and peer review

Not commissioned, externally peer-reviewed.

## Declaration of Competing Interest

Dr. Domenico Merlicco reported no biomedical financial interests or potential conflicts of interest.

Dr. Massimo Lombardi reported no biomedical financial interests or potential conflicts of interest.

Dr. Maria Carmela Fino reported no biomedical financial interests or potential conflicts of interest.
